# Gamma Irradiation Effects on Oxidation, Microbial Growth, and Volatile Compounds in Beef Aged at High Temperatures

**DOI:** 10.1021/acsomega.6c01595

**Published:** 2026-04-28

**Authors:** Lorena M. Rodrigues, Marielle Maria de O. Paula, Lorrany R. do Carmo, Paulo Rogério Fontes, Robledo A. Torres Filho, Alcinéia L.S. Ramos, Eduardo M. Ramos

**Affiliations:** † Department of Food Science, School of Agricultural Sciences of Lavras, Federal University of Lavras (UFLA), P.O. Box 3037, Lavras, Minas Gerais 37200-900, Brazil; ‡ Department of Food Technology, Federal University of Viçosa (UFV), Viçosa, Minas Gerais 36570-900, Brazil; § Institute of Exact and Technological Sciences, 549482 Federal University of Viçosa (UFV), Florestal Campus, Florestal, Minas Gerais 35690-000, Brazil

## Abstract

The present study aimed to evaluate the effects of gamma irradiation (0 and 3 kGy) and aging (up to 14 days) at different temperatures (1, 7, and 15 °C) on protein and lipid oxidation, mesophilic growth, and the volatile profile of bovine *Longissimus lumborum* muscle. Although irradiation has been proposed as a strategy to enable accelerated aging, limited information is available on its combined effects with elevated temperatures on the oxidative stability and volatile formation. Protein oxidation was not significantly affected (*P* > 0.05) by irradiation; however, carbonyl formation increased (*P* < 0.05) with longer aging periods and higher temperatures. Thiobarbituric acid reactive substance values ranged from 0.15 to 0.50 mg MDA/kg and were higher (*P* < 0.05) in irradiated samples aged for 14 days at elevated temperatures (7 and 15 °C). Mesophilic growth was greater (*P* < 0.05) in nonirradiated samples aged at higher temperatures, with mesophilic counts exceeding the acceptable limit (7 log CFU/g) after 7 days at 15 °C. Irradiation reduced (*P* < 0.05) mesophilic counts below the maximum acceptable limit (10^7^ CFU/g) across all treatments. Increased aging temperature significantly affected (*P* < 0.05) volatile formation, particularly those compounds associated with spoilage processes (lipid and protein oxidation and microbial metabolism), which were more abundant (*P* < 0.05) in nonirradiated samples stored at 15 °C. Irradiation had a limited effect on the volatile compounds formed, except for dimethyl sulfide, which increased in irradiated samples. Therefore, a low radiation dose can be effectively used to control mesophilic microbial growth under elevated aging temperatures, although its effect on lipid oxidation is dependent on storage conditions and may increase under extended aging at higher temperatures.

## Graphical abstract

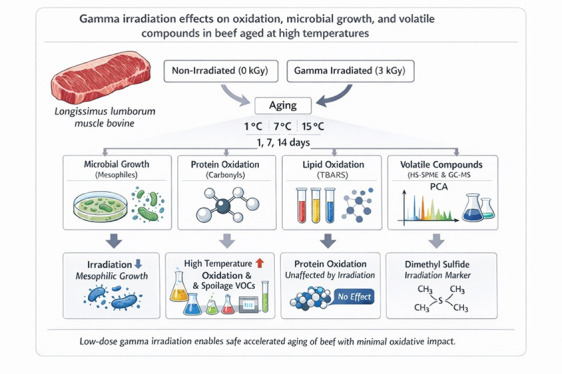

## Introduction

Tenderness and flavor are critical determinants of the consumer perception of beef quality, and consumers are generally willing to pay a premium for more tender meat. Among postmortem processing methods, aging is the most widely used technique in the meat industry to improve tenderness. The increase in tenderness and the development of small peptides and free amino acids (key contributors to the characteristic flavor of aged meat) result from the degradation of the myofibrillar structure by endogenous proteolytic enzymes.^1–3^

Conventionally, aging involves storing vacuum-packaged beef at temperatures close to 1 °C for approximately 3 weeks to enhance tenderness. However, this process requires substantial refrigerated storage capacity, energy, and operational costs.^4^ Increasing the aging temperature can accelerate tenderization by enhancing the activity of endogenous enzymes,^1^ thereby shortening the required aging period. Nevertheless, elevated temperatures also promote microbial proliferation, compromising meat shelf life,^5,6^ which limits the practical application of high-temperature aging. Therefore, shortening the tenderization time could become feasible if the microbial safety challenges associated with higher temperatures were overcome.

Irradiation has emerged as a potential strategy to control microbial growth during high-temperature meat aging. Some studies^6–10^ have evaluated the combined use of low doses (<5 kGy) of ionizing radiation (γ rays, electron beams, or X-rays) and elevated aging temperatures to accelerate postmortem tenderization. However, irradiation can affect meat quality by generating free radicals through water radiolysis, which can propagate lipid and protein oxidation and lead to the formation of volatile organic compounds (VOCs) responsible for the off-flavor and off-odor characteristics of irradiated meat.^11,12^

Among the VOCs derived from irradiation, sulfur compounds, such as dimethyl disulfide, produced from the radiolytic degradation of sulfur-containing amino acids (cysteine and methionine), are the primary contributors to the off-odor in irradiated meat.^13,14^ VOCs associated with undesirable sensory changes may also arise from lipid oxidation and microbial metabolism,^15,16^ both of which are enhanced at higher temperatures. Therefore, identifying and correlating VOCs in meat subjected to emerging processing technologies, such as high-temperature aging combined with irradiation, are essential for understanding their effects on product quality. Although previous studies have explored the use of irradiation to enable accelerated aging, most have focused primarily on tenderness and physicochemical traits with limited information available on the combined effects of irradiation and elevated aging temperatures on oxidative stability, microbial growth, and volatile compound formation. Accordingly, the objective of the present study was to evaluate the combined effects of a low γ radiation dose and different aging temperatures on protein and lipid oxidation, mesophilic growth, and the volatile organic compound profile of beef during aging. In this context, it was expected that low-dose irradiation (3 kGy) would control microbial growth under elevated aging temperatures, with a limited impact on oxidative stability and volatile compound formation.

## Materials and Methods

The animals used in the present work were the same as previously described by Rodrigues et al.^6^ Briefly, strip loins (*Longissimus lumborum*, LL) from different Nellore cattle (males of similar age, rearing system, and slaughter conditions) were obtained 48 h postmortem from a federally inspected slaughterhouse. The muscles were identified, vacuum-packaged, and transported in insulated boxes to the Meat and Meat Products Technology Laboratory (LabCarnes) of the Department of Food Science (DCA), Federal University of Lavras (UFLA).

### Preparation, Irradiation, and Aging of Samples

The irradiation dose of 3 kGy was selected based on previous studies demonstrating its effectiveness in reducing microbial load while minimizing adverse effects on meat quality, being considered a low-dose treatment.^6–10^ Aging temperatures were defined to represent different conditions: 1 °C as conventional refrigerated aging and 7 °C as a moderate temperature to simulate accelerated aging or mild temperature abuse, as previously reported.^5,7^ The temperature of 15 °C was included as an experimental condition to intensify biochemical and microbiological changes, allowing a better understanding of the combined effects of irradiation and elevated aging temperatures.

For this experiment, samples from three strip loins (from different animals) were used. Each strip loin was portioned into 18 steaks (experimental units, EUs) with a thickness of ∼1.5 cm. Steaks were individually weighed, vacuum-packaged (model BS420; R. Baião, Ubá, MG, Brazil) in nylon–polyethylene bags, randomly assigned to two groups (nonirradiated and irradiated) of nine EUs, and stored (at 2 °C) for 24 h before the radiation process. The refrigerated steaks of both groups were placed in insulated boxes with ice and transported to the Nuclear Technology Development Center of the National Nuclear Energy Commission (CDTN/CNEN) in Belo Horizonte, MG.

The box labeled “irradiated” was exposed to a γ radiation dose of 3 kGy using an IR-214 Gamma Cell Irradiator (MDS Nordion, Ottawa, Canada) equipped with a cobalt-60 source (dose rate: 1508.3 Gy/h). The second box, labeled “nonirradiated”, was kept at room temperature outside the irradiator for an equivalent period (∼119 min). Following irradiation, both boxes were returned to LabCarnes. Samples from each group were then randomized into three storage temperature treatments (1, 7, and 15 °C; three EUs per temperature) and stored in climate-controlled chambers (model EL202; EletroLab, São Paulo, Brazil). At each aging temperature, EUs were randomly sampled and analyzed after 1, 7, and 14 days of storage.

### Microbiological Analysis

For total mesophilic counts, portions of 25 g of meat were aseptically placed in sterile plastic bags containing 225 mL of sterile 0.1% peptone water and homogenized in a Stomacher (Metroterm, Brazil) at 490 strokes/min for 5 min. Serial decimal dilutions were prepared in sterile saline solution, and microorganisms were enumerated by the pour plate method using trypticase soy agar (TSA). Plates were incubated at 37 °C for 48 h, and the results were expressed as log colony-forming units per gram (log CFU/g).

### Protein Oxidation

Protein oxidation was assessed by quantifying carbonyl groups following derivatization with 2,4-dinitrophenylhydrazine (DNPH), according to Mercier et al.^17^ and Vossen and De Smet,^18^ with modifications. Approximately 2 g of meat was homogenized on ice (Turratec TE 102; TECNAL, Piracicaba, SP, Brazil) in 20 mL of phosphate buffer (20 mM, pH 6.5) containing 0.6 M NaCl and centrifuged (Hettich EBA21; Thermo Fisher Scientific, USA) at 3000×*g* for 10 min. Four aliquots (0.2 mL each) of the homogenate were transferred into Eppendorf tubes, mixed with 1 mL of 10% trichloroacetic acid (TCA), and kept on ice for 15 min to precipitate proteins. After centrifugation (3000×*g*, 10 min), the supernatants were discarded.

Pellets from two tubes were resuspended in 1 mL of 2.0 M HCl (blank), and pellets from the remaining two tubes were resuspended in 1 mL of 0.2% (w/v) DNPH prepared in 2.0 M HCl. The samples were vortexed, incubated for 1 h at room temperature (∼20 °C), and protected from light for derivatization. Protein was reprecipitated with 1 mL of 10% TCA, followed by centrifugation, and the resulting pellets were washed twice with 1 mL of ethanol:ethyl acetate (1:1, v/v). Pellets were then solubilized in 1.5 mL of phosphate buffer (20 mM, pH 6.5) containing 6.0 M guanidine HCl, vortexed, incubated for 30 min at room temperature, and centrifuged to remove insoluble residues.

The absorbance of the supernatant was measured at 370 nm using a spectrophotometer (Genesys 10 UV; Thermo Scientific Varian, São Paulo, Brazil), with phosphate buffer containing 6.0 M guanidine HCl as the blank. Protein concentration was determined by the Biuret method, and carbonyl content was expressed as nanomoles of DNPH per milligram of protein (nmol DNPH/mg), using a molar extinction coefficient (ε) of 22 mM^–1^·cm^–1^ for hydrazones at 370 nm.^18^

### Lipid Oxidation

Lipid oxidation was determined by the thiobarbituric acid reactive substance (TBARS) method, using an aqueous extraction procedure adapted from Pikul et al.^19^ Approximately 5 g of meat was homogenized (Turratec TE 102; TECNAL, Piracicaba, SP, Brazil) with 10 mL of distilled water and 0.5 mL of 4.2% butylated hydroxytoluene (BHT). Subsequently, 5 mL of 12% perchloric acid was added, and the mixture was vortexed and filtered. A 2 mL aliquot of the filtrate was combined with 2 mL of 0.08 M thiobarbituric acid (TBA) prepared in 3.86% perchloric acid, vortexed, and heated in a boiling water bath for 10 min. After being cooled on ice for 3 min, samples were centrifuged (Hettich EBA21; Thermo Fisher Scientific, USA) at 2200×*g* for 20 min.

The absorbance of the supernatant was measured at 532 nm using a spectrophotometer (Genesys 10 UV; Thermo Scientific Varian, São Paulo, Brazil), with 0.08 M TBA in 3.86% perchloric acid as the blank. TBARS values were expressed as milligrams of malondialdehyde (MDA) per kilogram of sample (mg MDA/kg), calculated from a calibration curve using 1,1,3,3-tetraethoxypropane (TEP) as the standard.

### Volatile Compound Profile

Separation and identification of volatile compounds were carried out at the Central Laboratory of Chemical Analysis and Prospecting (CAPQ), Department of Chemistry, Federal University of Lavras (UFLA), using a gas chromatograph coupled to a mass spectrometer (GC–MS QP2010 Plus; Shimadzu, Kyoto, Japan). The system was equipped with an automatic liquid/gas sampler (AOC-5000; Shimadzu, Kyoto, Japan) and an SLB-5MS capillary column (5% phenyl–95% dimethylpolysiloxane; 30 m × 0.25 mm i.d., 0.25 μm film thickness; Supelco, Bellefonte, PA, USA). Extraction and analysis were conducted according to Paula et al.,^14^ with minor modifications.

Volatile analysis was conducted to verify the combined effects of irradiation and maturation temperature. Thus, portions from the three steaks of each aging time (1, 7, and 14 days) were minced together to obtain a composite sample. Approximately 2.5 g of the sample was placed in a 22 mL vial sealed with a silicone/PTFE septum (Supelco, Bellefonte, PA, USA). Volatile organic compounds were extracted by headspace solid-phase microextraction (HS-SPME) using a DVB/CAR/PDMS fiber (10 mm length, 50–30 μm thickness; Supelco, Bellefonte, PA, USA). A blank test was conducted to recondition and verify the fiber cleanliness and background noise level from the septa used in the sample vials. Before each extraction, the fiber was conditioned at 250 °C for 5 min to avoid carryover composites. The vials were preheated at 60 °C for 10 min, and the fibers were then exposed to the headspace for 45 min. Thermal desorption was performed in a splitless mode at 250 °C for 5 min. Helium (99.999%) was used as the carrier gas at a flow rate of 1.0 mL/min. The oven temperature program started at 35 °C (held for 2 min), increased at 2 °C/min to 80 °C, at 4 °C/min to 150 °C, and at 8 °C/min to 230 °C. Mass spectrometry parameters were as follows: ionization energy, 70 eV; scan range, 45–350 Da; solvent cutoff, 0.55 min; interface temperature, 250 °C; and ion source temperature, 200 °C.

Compounds were identified by comparing their mass spectra with those in the NIST libraries (Wiley 8 and FFNSC 1.2) and by matching the Kovats index (KI) with literature values (difference equal to or less than 10), using a homologous series of alkanes (Supelco, Bellefonte, PA, USA). The identifications of some volatile compounds were performed only by using mass spectrometry data because the retention index was unavailable. Peak areas were integrated using GCMS Solution software (Shimadzu, Kyoto, Japan), and data were expressed as total ion counts and should be considered semiquantitative, representing the relative abundance of volatile compounds.

### Statistical Analysis

The experiment was arranged in a randomized block design (RBD), with animals as blocks (three repetitions). Microbiological, protein, and lipid oxidation analyses were evaluated in a split-plot scheme, with irradiation (0 and 3 kGy) × aging temperature (1, 7, and 15 °C) as main plots and aging time (1, 7, and 14 days) as subplots. Volatile organic compounds (VOCs) were evaluated in a factorial design: irradiation (0 and 3 kGy) × aging temperature (1, 7, and 15 °C), with aging time (combined sample) as a replicate. Main and interaction effects were assessed by analysis of variance (ANOVA) at a 5% significance level. Tukey’s test and Duncan’s test (for VOCs) were applied when necessary to separate means (*P* < 0.05). Analyses were performed using Statistica software, version 8.0 (StatSoft Inc., Tulsa, USA).

Principal component analysis (PCA) was performed with Sensomaker software, version 1.91 (UFLA, Brazil), to explore VOC patterns over aging time and to associate the set of samples with chemical and microbiological attributes. Before PCA, VOCs were transformed using log_10_ to normalize the data and favor the separation between groups.^20^ They were also mean-centered and autoscaled (unit variance scaling) to ensure that all variables had a mean of zero and a variance of one.

## Results and Discussion

### Total Mesophilic Counts

A significant three-way interaction (*P* = 0.002) was observed among irradiation, temperature, and aging time for total mesophilic counts, and the interaction effects are presented in Figure 1. In irradiated meat aged at the conventional temperature (1 °C), total mesophilic counts remained below the detection limit (1.0 log CFU/g) at all aging times. At elevated temperatures (7 and 15 °C), counts were also below this limit after 1 day of aging; however, microbial recovery was observed thereafter. This regrowth can be attributed to the sublethal damage induced by irradiation. Ionizing radiation does not necessarily cause immediate cell death in all microorganisms; instead, it can induce sublethal injury to DNA, cell membranes, and enzymatic systems. Under such conditions, injured cells may remain temporarily inactive but retain the ability to repair damage through cellular mechanisms, such as recombinational repair and the SOS response. When subsequently exposed to favorable environmental conditions, such as elevated storage temperatures, these repair processes are enhanced, allowing bacterial cells to recover and resume proliferation. This phenomenon has been widely reported in irradiated meat systems, where higher temperatures accelerate metabolic activity and promote the outgrowth of sublethally injured populations.^6,21^

**Figure 1:**
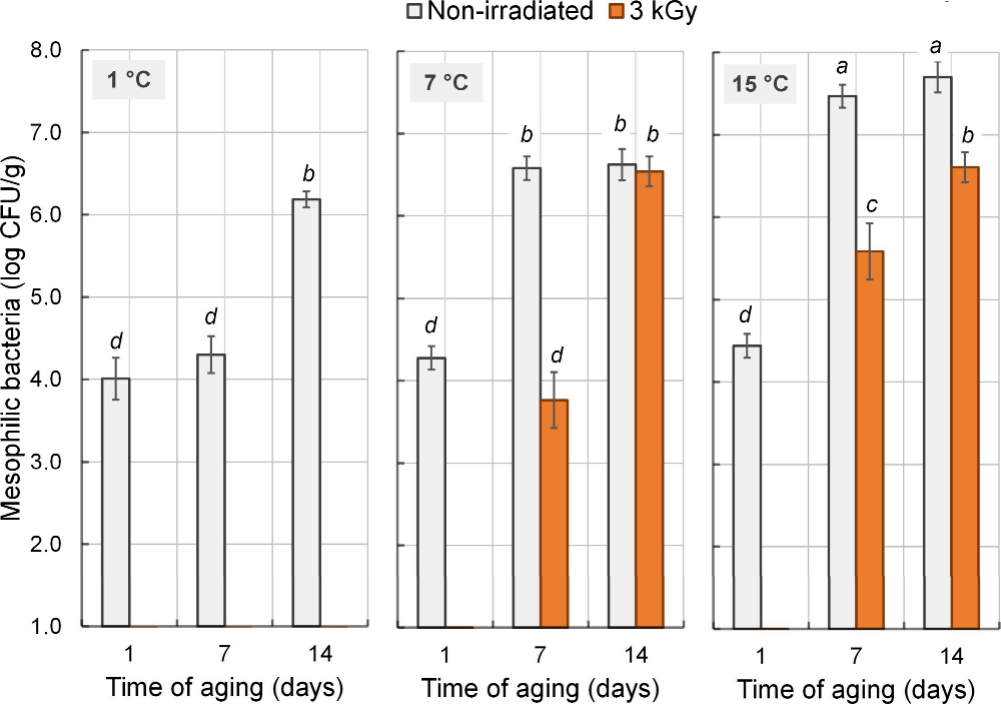
Effects of γ radiation (3 kGy) and aging temperature (1, 7, and 15 °C) on mesophilic bacterial counts (a limit of detection of 1.0 log CFU/g) in strip loin beef during the aging period. Error bars represent the standard error of the mean (*n* = 3). ^a–d^ Means followed by different letters differ (*P* < 0.05) by Tukey’s test.

Nonirradiated meat exhibited significantly higher (*P* < 0.05) mesophilic counts than irradiated samples, regardless of aging temperature, except for those aged at 7 °C for 14 days. Notably, irradiation maintained mesophilic counts below the acceptability threshold (7 log CFU/g) in strip loins aged at 15 °C for 14 days, maintaining counts below the acceptability threshold of 7 log CFU/g for fresh meat,^22^ whereas nonirradiated samples exceeded this limit after only 7 days of aging.

The inhibitory effect of irradiation and the stimulatory influence of higher aging temperatures on microbial growth have also been reported previously.^4,6–8,10^ The antimicrobial effects of irradiation occur either directly, through the absorption of radiation energy by microbial cells, or indirectly, via the formation of free radicals and hydrogen peroxide (H_2_O_2_) resulting from the radiolysis of water molecules. These reactive species can damage bacterial cell walls and DNA, leading to structural dysfunction and cell death.^23^ However, elevated aging temperatures favor microbial growth, facilitating the recovery and proliferation of surviving microorganisms,^5^ even in irradiated samples.

However, it is important to note that these results are limited to total mesophilic counts and do not allow conclusions regarding microbial safety, as no specific pathogens or broader spoilage microbiota were evaluated.

### Protein and Lipid Oxidation

Protein oxidation was not affected (*P* > 0.05) by irradiation or any interaction among the evaluated factors. In contrast, lipid oxidation showed a different response pattern, being significantly influenced by irradiation under specific conditions, particularly at higher temperatures and extended aging periods. An increase in carbonyl content was expected in irradiated meat since most reactive species (free radicals—hydroxyl, OH^·^; superoxide, O_2_^·^^–^ and nonradicals—hydrogen peroxide, H_2_O_2_) known to initiate protein oxidation are generated through water radiolysis.^24,25^ Conversely, Zhang et al.^26^ reported that irradiation at 3 kGy increased total carbonyl content in pork stored for 14 days at 4 °C but did not affect sulfhydryl groups. Similarly, Rowe et al.^27^ observed higher carbonyl content in beef irradiated at 6.4 kGy after 7 days of aging at 4 °C.

The vacuum packaging conditions used in this study likely played an important role in modulating the oxidative processes. The reduced oxygen availability under vacuum limits the formation of oxygen-dependent reactive species, which may contribute to the observed low level of protein oxidation. However, lipid oxidation can still occur under these conditions through radical-mediated mechanisms initiated by irradiation or endogenous pro-oxidants, particularly at higher temperatures and extended aging times. This may explain the different responses observed for protein and lipid oxidation in the present study.

Protein oxidation was affected (*P* > 0.05) by time and temperature of aging individually. Higher (*P* < 0.05) carbonyl content was observed in meat aged at 15 °C compared to the conventional temperature (1 °C), which did not differ (*P* > 0.05) from meat aged at 7 °C (Figure 2A). Moreover, carbonyl content increased (*P* < 0.05) with aging time, being higher (*P* < 0.05) in meat aged for 14 days than in meat aged for 7 days, which in turn was higher (*P* < 0.05) than in meat aged for only 1 day (Figure 2B). Carbonyl formation is one of the most relevant alterations in oxidized muscle proteins, accumulating in beef during aging,^3,28^ as observed in the present study.

**Figure 2:**
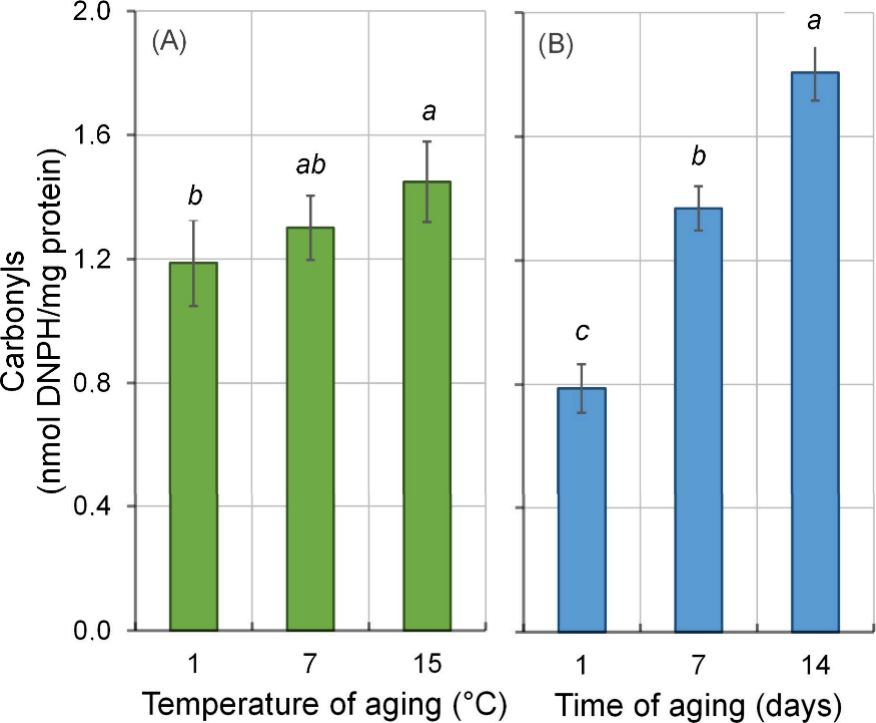
Effects of (A) temperature and (B) time of aging on carbonyl content in strip loin beef during the aging period. Error bars represent the standard error of the mean (*n* = 9). ^a–c^ Means followed by different letters, within time or temperature of aging, differ (*P* < 0.05) by Tukey’s test.

Different from protein oxidation, lipid oxidation was significantly affected (*P* < 0.05) by the three-way interaction among irradiation and temperature and time of aging. In all samples, lipid oxidation, indicated by higher TBARS values, increased (*P* < 0.05) throughout the aging period, being more pronounced in irradiated meat aged at 7 and 15 °C (Figure 3). In nonirradiated meat, aging at 15 °C promoted greater TBARS formation compared with 1 and 7 °C, which did not differ. Moreover, TBARS values were higher (*P* < 0.05) in most irradiated samples compared with their nonirradiated counterparts. Lower temperatures slow lipid oxidation in meat, whereas higher temperatures accelerate this process.^29^ Reactive oxygen species (ROS) formed during aging or generated by irradiation can react with lipids, enhancing oxidative processes.^30^ Irradiation can also alter the redox potential of meat, thereby promoting lipid oxidation, which may lead to undesirable sensory and quality effects, such as off-flavors, off-odors, discoloration, and nutrient loss.^12,25^

**Figure 3:**
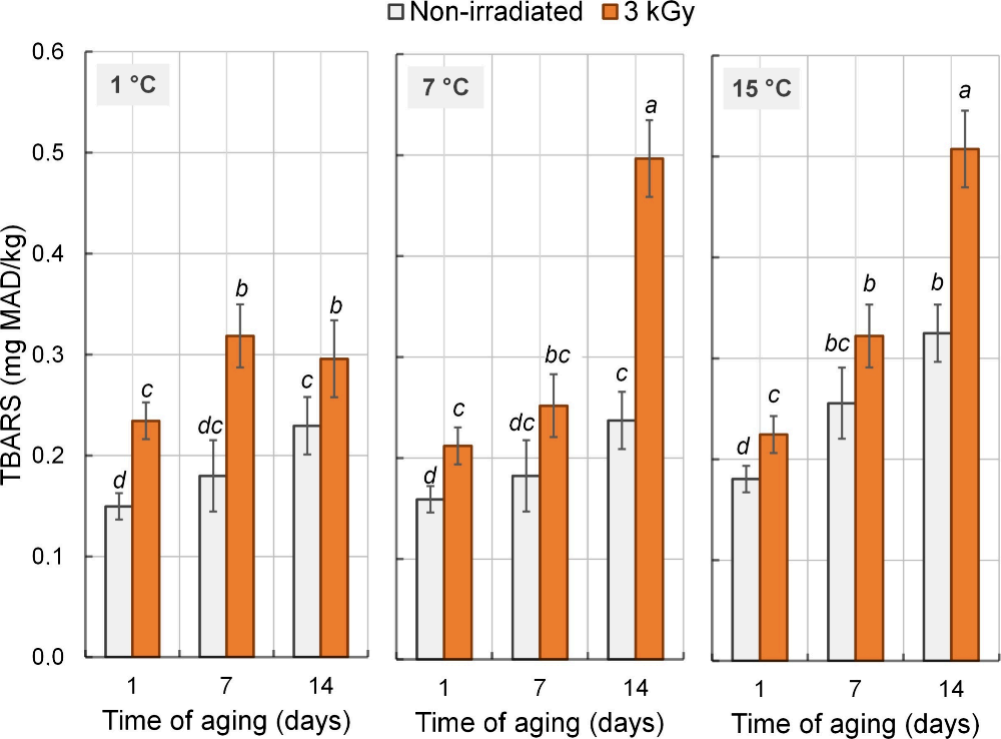
Effects of γ radiation (3 kGy) and aging temperature (1, 7, and 15 °C) on TBARS values in strip loin beef during the aging period. Error bars represent the standard error of the mean (*n* = 3). ^a–d^ Means followed by different letters differ (*P* < 0.05) by Tukey’s test.

Vitale et al.^31^ reported that TBARS values in beef aged at 1 and 7 °C did not differ significantly across aging times (up to 21 days), although oxidation increased over extended periods, particularly beyond 21 days. Similarly, Chen et al.^32^ observed an increase in TBARS in bovine *Semitendinosus* muscles packaged in oxygen-permeable films with increasing irradiation dose (0–3.17 kGy) on day 0. However, after 10 days of storage at 7 °C, the TBARS values were lower in irradiated samples. Rodrigues et al.^4^ also reported higher TBARS in vacuum-packed *Longissimus lumborum* muscles exposed to 9 kGy but not at doses of 3 or 6 kGy. According to these authors, the packaging atmosphere plays a critical role in modulating oxidative reactions in lipid oxidation compared to irradiation itself, as differences between irradiated and nonirradiated meat become more pronounced when irradiation occurs in the presence of oxygen. In contrast, under vacuum conditions, the pro-oxidant effects of irradiation are markedly reduced.^33^

Under the conditions of this study, lipid oxidation was more responsive to irradiation than protein oxidation. Nevertheless, the effect of irradiation on lipid oxidation was dependent on aging conditions, with increases observed particularly at higher temperatures and longer aging times, except in meat aged at higher temperatures for 14 days. In the present work, TBARS values (0.15–0.32 mg MDA/kg) in meat aged at 1 °C for up to 14 days were lower than those (0.21–0.39 mg MDA/kg) reported in ref 4 in meat irradiated up to 6 kGy under similar conditions. Furthermore, TBARS values observed by these authors in meat irradiated at 9 kGy (0.87 mg of MDA/kg) exceeded those found here (∼0.50 mg of MDA/kg) in meat irradiated and aged at higher temperatures for 14 days.

However, it is important to note that the TBARS values obtained in this study are well below the threshold reported by Campo et al.,^34^ at which rancid flavor becomes sensorially dominant. These authors identified a threshold of 2.0 mg MDA/kg using the distillation extraction method, which, according to Pikul et al.,^19^ yields TBARS values approximately 1.35 times higher than those obtained by aqueous extraction (as applied in the present study) corresponding to an equivalent threshold of approximately 1.5 mg MDA/kg.

### Volatile Compounds

The evaluation of volatile organic compounds (VOCs) enabled the identification of 23 compounds (Table 1), classified into the following groups: alcohols (*n* = 9), aldehydes (*n* = 8), ketones (*n* = 2), hydrocarbons (*n* = 3), and sulfur compounds (*n* = 1).

**Table 1:** Effects of Irradiation (*I*) and Aging Temperature (*T*) on the Volatile Compound Profile (Total Ion Counts × 10^3^) of Strip Loin Beef^a^

			irradiation (kGy)		aging temperature (°C)				Pr > *F* ^1^		
groups/compounds	KI_C_	KI_L_	0	3	1	7	15	SEM	*I*	*T*	*I* × *T*
alcohols											
1-octen-3-ol	979	974	457^x^	38^y^	6^b^	14^b^	722^a^	105	**0.045**	**0.019**	0.074
>2,3-butanediol	806	785	201^x^	28^y^	16	232	95	59	**0.038**	0.706	0.950
2-ethylhexanol	1029		13	31	29	21	16	6	0.239	0.761	0.647
benzeneethanol	1110	1106	35	21	/	13	70	13	0.857	0.622	0.427
ethanol	491	500	363	214	100^b^	260^ab^	506^a^	69	0.136	**0.021**	0.439
heptanol	970	959	56^x^	2^y^	/	7^b^	81^a^	19	**0.046**	**0.050**	0.082
hexanol	869	863	245^x^	2^y^	/	3^b^	367^a^	86	**0.047**	**0.028**	0.102
nonanol	1172	1165	64	13	/	16^b^	98^a^	18	0.134	**0.021**	0.222
octanol	1073	1063	215	172	172	156	254	21	0.176	**0.021**	**0.044**
aldehydes											
benzaldehyde	957	952	1061	193	88^b^	181^b^	1611^a^	322	0.154	**0.009**	0.214
benzeneacetaldehyde	1040	1036	491	121	11^b^	357^ab^	550^a^	130	0.932	**0.039**	0.315
decanal	1202	1201	29	6	13	7	33	6	0.746	0.131	0.512
heptanal	902	901	128	34	36^b^	24^b^	183^a^	31	0.072	**0.004**	0.225
hexanal	802	801	399^x^	77^y^	62^b^	48^b^	605^a^	115	**0.049**	**0.013**	0.145
nonanal	1102	1100	1428	1026	994^b^	877^b^	1811^a^	151	0.331	**0.001**	0.434
octanal	1001	998	203	125	122^b^	107^b^	263^a^	27	0.211	**0.002**	0.440
tetradecanal	1810		393	294	146^b^	310^ab^	575^a^	58	0.691	**0.007**	0.700
ketones											
2-propanone	505		8	9	6	10	10	3	0.977	0.808	0.424
3-hydroxy-2-butanone (acetoin)	711	711	152	297	224	376	74	96	0.951	0.414	0.648
hydrocarbons											
hexane	602	600	1406	1955	1613	1848	1582	226	0.112	0.628	0.299
methylbenzene (toluene)	663		985	1489	1376	1284	1050	209	0.085	0.623	0.471
octane	798	800	2	7	5	1	6	2	0.075	0.327	0.571
sulfurous											
dimethyl sulfide	512	510	1	79	20	40	59	9	***<0.001***	**0.048**	**0.047**
Σ alcohols			1649^x^	521^y^	323^c^	722^b^	2209^a^	373	**0.046**	**0.018**	0.160
Σ aldehydes			4132^x^	1876^y^	1472^b^	1911^b^	5631^a^	354	**0.044**	**0.011**	0..148
Σ ketones			160	306	230	386	84	96	0.467	0.473	0.834
Σ hydrocarbons			2393	3451	2994	3133	2638	437	0.759	0.526	0.129
Σ sulfurous			1	79	20	40	59	9	0.459	0.592	0.924
total			8335	6233	5039	6192	10,621				

a KI_C_ = Kovats index calculated; KI_L_ = Kovats index from the literature; SEM = standard error of the mean (*n* = 12); / = not detected. ^1^Significant probabilities (*P* < 0.10) are highlighted in bold. Means followed by different letters in the row, ^x,y^ for the radiation doses and ^a–c^ for the aging temperature, differ (*P* < 0.05) by the F-test and Duncan’s test, respectively.

Regarding these groups, significant effects (*P* < 0.05) of irradiation and aging temperature were observed for alcohols, while only aging temperature affected aldehydes. Irradiated meat exhibited lower (*P* < 0.05) concentrations of alcohols primarily due to reduced levels of 1-octen-3-ol, 2,3-butanediol, heptanol, and hexanol.

Alcohols, like ketones, have low odor thresholds,^35^ making them important contributors to meat flavor. Alcohols such as 1-octen-3-ol, hexanol, and 2,3-butanediol are associated with both lipid oxidation and microbial metabolism.^16,36^ In the present study, their lower abundance in irradiated meat is consistent with reduced microbial activity, as also reflected in mesophilic counts (Figure 1), while their increase at higher temperatures (15 °C) indicates enhanced oxidation and bacterial growth, in agreement with previous reports.^14,37,38^

Among the identified alcohols, only octanol was significantly affected (*P* < 0.05) by the irradiation × aging temperature interaction (Table 1), being more abundant in nonirradiated meat aged at 15 °C (Figure 4A). This increase may be attributed to greater microbial proliferation at this temperature, as previously associated with spoilage microbiota such as *B. thermosphacta*.^39^

**Figure 4:**
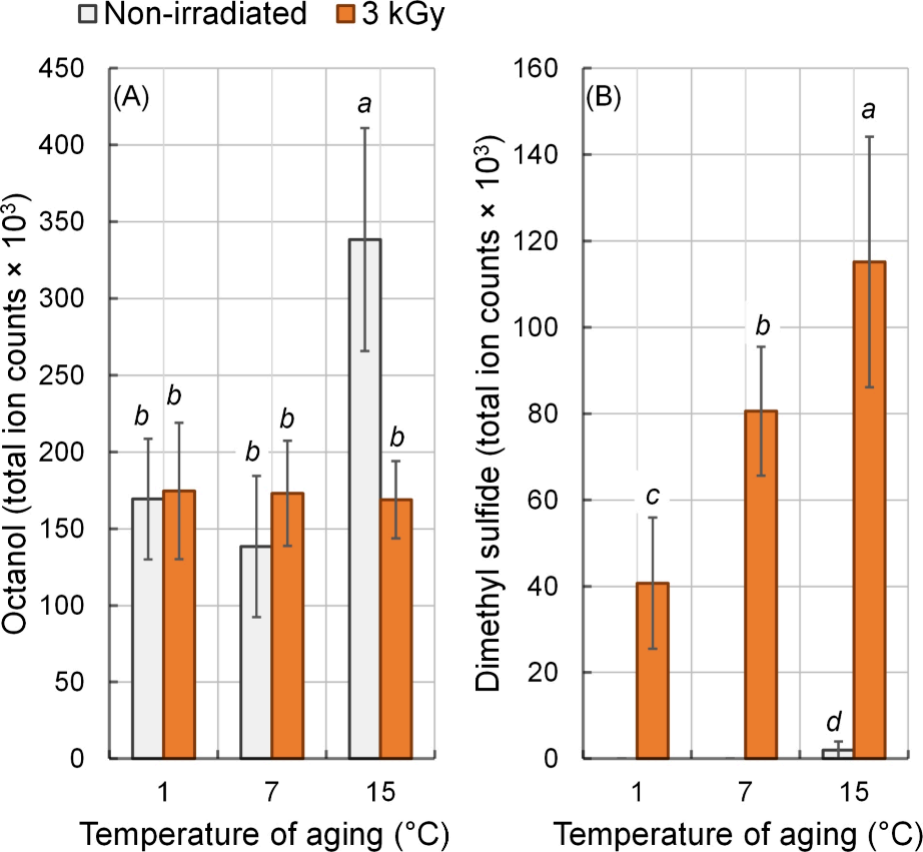
Effects of γ radiation (3 kGy) on the formation of (A) octanol and (B) dimethyl sulfide compounds in strip loin beef aged at different temperatures (1, 7, and 15 °C). Error bars represent the standard error of the mean (*n* = 3). ^a–d^ Means followed by different letters differ (*P* < 0.05) by Tukey’s test.

Hexanal was the only aldehyde significantly affected (*P* < 0.05) by irradiation. Hexanal originates from the auto-oxidation of linoleic acid and is widely recognized as a reliable indicator of lipid oxidation in meat.^40^ However, its formation may also be influenced by microbial activity, which explains the higher levels observed in nonirradiated meat and in samples aged at higher temperatures.

Although aldehydes are primarily derived from lipid oxidation, their formation may also be influenced by microbial activity.^16^ The higher levels observed in meat aged at 15 °C indicate intensified oxidative reactions and microbial proliferation, while their reduction in irradiated samples is consistent with microbial control.^25,40^

Ketones and hydrocarbons were not significantly affected (*P* > 0.05) by irradiation or aging temperature, either individually or as groups. Similar to aldehydes, these compounds can also arise from fatty acid oxidation and contribute to meat flavor.^40^ Paula et al.^14^ reported that the main irradiation-related change among ketones (3–9 kGy) was the reduced presence of 3-hydroxy-2-butanone (acetoin), which occurred at much higher concentrations in nonirradiated meat. However, this reduction was not significant (*P* > 0.05) in the present study. Some aromatic hydrocarbons, such as methylbenzene (toluene), may naturally occur in meat due to dietary sources or be produced in ruminant meat by ruminal microorganisms.^16^

The absence (*P* > 0.05) of irradiation effects on total ketones and aldehydes aligns with the lack of effect (*P* > 0.05) on protein carbonyl content measured by the DNPH assay. Likewise, the increase (*P* < 0.05) in carbonyl content at higher aging temperatures may be attributed to the corresponding rise (*P* < 0.05) in total aldehyde concentration.

A significant (*P* < 0.05) irradiation × temperature interaction was observed for dimethyl sulfide levels (Figure 4B). Higher concentrations of dimethyl sulfide were found in irradiated meat aged at elevated temperatures (7 and 15 °C), whereas, in nonirradiated meat, this compound was detected only in trace amounts at 15 °C. It should be noted that compound identification was based on mass spectral library matching and retention indices, without confirmation using analytical standards, which limits its interpretation as a specific irradiation marker. Thus, dimethyl sulfide should be interpreted as a compound associated with irradiation under the evaluated conditions rather than a definitive marker. Similar observations have been reported in previous studies, where sulfur compounds, including dimethyl sulfide, were consistently detected in irradiated meat and other irradiated food systems as a result of radiolytic degradation of sulfur-containing amino acids.^13,25^ However, these compounds are not exclusive to irradiation, as they may also be formed through microbial metabolism or thermal and oxidative reactions. Therefore, their use as irradiation biomarkers should be interpreted with caution as they lack specificity and may not be universally applicable across different food matrices and processing conditions.

The importance of dimethyl sulfide lies in the established role of sulfur compounds as key contributors to irradiation odor.^13^ These compounds are formed through the radiolytic degradation of sulfur-containing amino acids (cysteine and, primarily, methionine). According to Brewer,^25^ the generation of such compounds indicates that radiolytic degradation of sulfur-containing proteins contributes more to off-odor formation than lipid oxidation. Even trace amounts of these volatiles can impart the characteristic irradiation odor due to their extremely low odor thresholds.^41^ Therefore, irradiated meat may develop a characteristic irradiation odor depending on the extent of radiolytic reactions.

Odors described as “cabbage”, “sulfur”, “spoiled vegetable”, or “roasted corn” may arise depending on the type and extent of radiolytic degradation of sulfur compounds.^25^ However, such compounds can also result from protein oxidation caused by factors other than irradiation. Paula et al.^14^ reported that although dimethyl sulfide was initially detected only in irradiated samples, it was also found in nonirradiated meat after 14 days of aging. Nonetheless, dimethyl sulfide levels were approximately 1.5 times higher in irradiated meat and were more strongly associated with higher irradiation doses (6 and 9 kGy). Similarly, O’Quinn et al.^42^ detected substantial dimethyl sulfide accumulation in beef aged for 46 days, noting that its concentration was positively correlated with undesirable bloody/metallic and bitter flavors and negatively correlated with desirable attributes, such as beefy/brothy, browned/grilled, buttery/beef fat, nutty/roasted nut, and sweet flavors, thereby reducing consumer acceptance.

In addition to its radiolytic origin, dimethyl sulfide formation at elevated aging temperatures may also result from microbial metabolism. According to Casaburi et al.,^16^ microbial degradation of meat can generate several sulfur compounds, including dimethyl sulfide (identified in the present study) as well as dimethyl disulfide and dimethyl trisulfide. These authors also associated dimethyl sulfide production with spoilage in vacuum-packed meat contaminated by *B. thermosphacta*.

Overall, the identified volatile compounds can be broadly associated with two main pathways: lipid oxidation (mainly aldehydes) and microbial metabolism (mainly alcohols and sulfur compounds).

A principal component analysis (PCA) was performed using individual volatile compounds to illustrate differences among treatments throughout the aging period and to evaluate their relationships with oxidative and microbiological parameters (Figure 5). The first two principal components explained 57.35% of the total variance, with PC1 and PC2 accounting for 39.21 and 18.14%, respectively. Meat aged for 1 day, regardless of irradiation or aging temperature, was positioned in the lower-left quadrant, opposite lipid (TBARS) and protein (CARB) oxidation and mesophilic counts (TMC) located in the upper-right quadrant. This distribution indicates a weak association between early aged samples and oxidative or microbiological changes.

**Figure 5:**
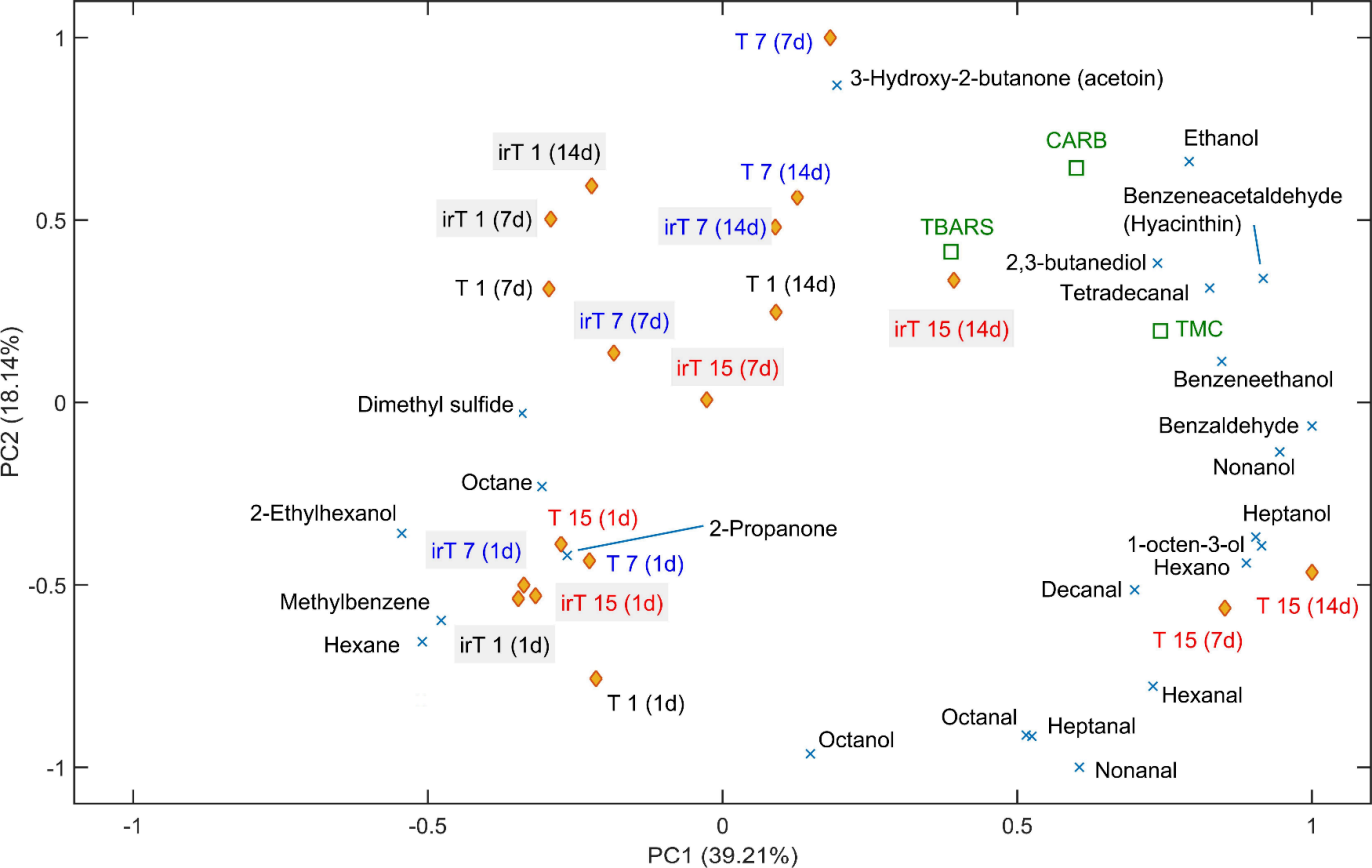
Principal component analysis (PCA) plot of volatile compounds, lipid (TBARS) and protein (CARB) oxidation values, and mesophile counts (TMC) in strip loin beef either subjected or not to 3 kGy γ radiation (“ir”, gray-colored) and aged at different temperatures (“*T*” = 1, 7, and 15 °C) for 1, 7, and 14 days (“d”, in parentheses).

In contrast, meat aged for longer periods was more strongly associated with these parameters, particularly at 15 °C. Except for 2-ethylhexanol, most alcohols were located on the right side of PC1, near mesophilic counts, and were primarily associated with nonirradiated samples aged at 15 °C for 7 and 14 days. The compound dimethyl sulfide was positioned close to most irradiated samples, but in the opposite quadrant, compounds were associated with oxidative and microbiological changes. This distribution confirms that low irradiation exerted relatively minor effects on lipid and protein oxidation, whereas these processes were more strongly influenced by postmortem proteolysis and microbial activity during aging, particularly at elevated temperatures (15 °C).

## Conclusions

Gamma irradiation (3 kGy) effectively controlled mesophilic microbial growth during aging at elevated temperatures for up to 7 days based on the evaluated parameters. Its effect on lipid oxidation was condition-dependent, with increases observed at higher temperatures and longer aging times, while protein oxidation was not affected. Increasing aging temperature, particularly to 15 °C, accelerated oxidative processes and microbial growth in nonirradiated samples. In contrast, irradiation had a limited impact on volatile compound formation compared with temperature effects.

The combination of irradiation with an aging temperature of 7 °C may represent a strategy to control microbial growth while limiting protein oxidation under elevated aging conditions. However, this study has some limitations, including the relatively small biological sample size, the pooled design used for volatile compound analysis, the absence of sensory evaluation, and the lack of pathogen-level microbiological data. Further studies addressing these aspects are required to confirm the practical applicability of these conditions.
